# Cytomegalovirus infection in heart transplant recipients: Epidemiology, risk factors, and long-term outcomes from a major transplant center in the United States

**DOI:** 10.1016/j.jhlto.2023.100047

**Published:** 2023-12-22

**Authors:** Bismarck S. Bisono-Garcia, Zachary A. Yetmar, Vaisak Nair, Lisa Brumble, Holenarasipur R. Vikram, Raymund R. Razonable, Elena Beam

**Affiliations:** a Division of Public Health, Infectious Diseases and Occupational Medicine. Mayo Clinic, Rochester, Minnesota; b Department of Infectious Disease, Cleveland Clinic Foundation, Cleveland, Ohio; c Department of Infectious Diseases, Mayo Clinic, Jacksonville, Florida; d Division of Infectious Diseases, Mayo Clinic, Phoenix, Arizona

**Keywords:** cytomegalovirus, heart transplantation, opportunistic infection, antiviral

## Abstract

**Background:**

Despite the use of antiviral prophylaxis, cytomegalovirus (CMV) remains a common opportunistic infection following heart transplantation. This study analyzes the rates, risk factors, and outcomes of CMV among heart transplant recipients.

**Methods:**

A retrospective cohort study was conducted of adults who underwent heart transplantation between January 1, 2011, and March 31, 2019. The primary outcome was clinically significant CMV infection (csCMVi), defined as CMV disease or asymptomatic infection requiring pre-emptive therapy. The secondary outcome was all-cause mortality. Patients received valganciclovir prophylaxis up to 6 months, depending on CMV donor/recipient serostatus. Kaplan-Meier curve and multivariable Cox regression were used for outcome analysis.

**Results:**

Among 553 heart transplant recipients, 101 (18.3%) experienced csCMVi, including 35 (6.3%) with CMV disease. csCMVi was uncommon during prophylaxis. In multivariable analysis, CMV D+/R– status hazard ratio (HR 12.88, 95% CI 6.76-24.56; *p* < 0.001) and lower absolute lymphocyte counts in seropositive recipients (HR 1.48, 95% CI 1.23-1.79; *p* < 0.001), but not CMV D+/R– patients (HR 1.18, 95% CI 0.94-1.47; *p* = 0.162), were significantly associated with csCMVi. Sixty patients died during follow-up, and csCMVi was associated with increased mortality (HR 2.84, 95% CI 1.62-4.98; *p* < 0.001).

**Conclusions:**

In this large cohort of heart transplant recipients, csCMVi was linked to higher mortality. CMV D+/R– serostatus was associated with an increased risk of csCMVi, with lower absolute lymphocyte counts increasing risk only in CMV seropositive recipients. Strategies for optimizing CMV prevention in serodiscordant heart recipients are warranted.

## Background

Cytomegalovirus (CMV) is a herpesvirus that commonly affects immunocompromised patients, including heart transplant recipients.^1^ CMV can cause an asymptomatic infection or manifest with clinical symptoms, including organ-invasive CMV disease.^2^ Despite advances in transplantation medicine, CMV infection continues to be a challenge for transplant patients due to the ability of this virus to reactivate when the immune status of the host is overly suppressed.

Incidence of CMV varies among transplant recipients, depending on factors such as the transplanted organ and the CMV serostatus of the donor (D) and recipient (R). The highest risk is observed when the donor is CMV serostatus positive and the recipient is CMV negative (D+/R–),^3^ with over half of these patients experiencing CMV infection and about one-third with CMV disease.^4^, ^5^ CMV reactivation risk is further influenced by numerous factors, such as the transplant recipient’s overall immune status, as reflected by lymphocyte count and immunosuppressive drug levels, and intrinsic viral characteristics.^6^, ^7^, ^8^, ^9^ The immune system plays a key role on the reactivation of CMV and the degree of disease, with the presence of CMV-specific CD8+ T-cells linked to protection against CMV.^10^ This protection may be reduced when using T-cell depleting agents for induction immunosuppression at the time of transplant, post-transplant, or intensified immunosuppression for rejection episode treatment.

CMV infection or reactivation in heart transplant recipients is a major cause of morbidity and mortality in this population.^3^, ^11^, ^12^, ^13^ However, while past studies have identified risk factors, there have been many changes in transplantation practices and there is a need for more contemporary data. Thus, we aimed to estimate the incidence of CMV infection after heart transplantation and analyze proposed risk factors in a modern era. Additionally, we sought to analyze long-term outcomes, primarily post-transplant mortality after development of CMV infection.

## Methods

### Study design and setting

We performed a multicenter, retrospective cohort study of adult heart transplant patients at the Mayo Clinic Transplant Center, with sites located in Arizona, Florida, and Minnesota. All 3 Mayo Clinic sites share common standardized transplantation protocols through the study period, including the use of antiviral prophylaxis post-transplantation. There was no significant change in protocols during the study period, and CMV testing was performed across all sites using the same CMV PCR platform (COBAS AmpliPrep/COBAS Taqman CMV test prior to 2018, and thereafter, COBAS 6800/8800 assay, Roche Diagnostics). CMV D+/R– patients received valganciclovir 900 mg once daily for 6 months post-transplant, while CMV R+ patients received valganciclovir 900 mg once daily for 3 months post-transplant; all drug doses were adjusted based on renal function. If prophylaxis was discontinued early, patients were monitored once weekly using CMV PCR test. In addition, CMV PCR testing was performed once weekly for 12 weeks after completion of 6 months of prophylaxis among CMV D+/R– heart recipients. Patients were educated on the signs and symptoms of CMV infection and were advised to alert their transplant team if these occur. Treatment of CMV infection and disease was individualized. However, our institution has general guidance to initiate antiviral treatment if patients have suspected symptomatic infection (i.e., CMV syndrome or tissue-invasive disease), if asymptomatic but their plasma CMV DNA level is >1000 IU/ml, or if their plasma CMV DNA level is below this level but is increasing on serial once weekly testing. Any viral load level in CMV D+/R– heart recipients was generally treated. CMV D–/R– patients received acyclovir 400 mg twice daily for 1-month post-transplant for the prevention of herpes simplex virus infection. For patients who received augmented immunosuppression for acute rejection, D+/R– and R+ recipients received either 1 or 3 months of valganciclovir prophylaxis if they received pulse-dose corticosteroids or lymphocyte-depleting agents, respectively. Induction immunosuppression was typically either basiliximab or antithymocyte globulin, based on immunologic and infectious risk factors. Standard post-transplant immunosuppression included tacrolimus, mycophenolate mofetil, and prednisone tapered to 5 mg once daily. Our institutional review board reviewed the study protocol and granted it an exempt status (#19-000302).

### Inclusion and exclusion criteria

Heart transplant recipients who underwent transplantation from January 1, 2011, through March 31, 2019, were identified from our internal transplant center registry and assessed for inclusion. Inclusion criteria were ≥18 years of age on the date of transplantation with at least 3 months of post-transplant follow-up. Exclusion criteria were death or loss to follow-up prior to 3 months or lack of research authorization per Minnesota state statute. If patients underwent multiple heart transplant procedures during the study period, only the most recent transplant episode was included. Once eligible patients were identified, data were manually extracted from the electronic medical record. This included demographics, pretransplant characteristics, peri-transplant course, and outcomes. Absolute lymphocyte counts (ALCs) during the follow-up period were extracted from an internal laboratory database. Study data were collected and managed using REDCap electronic data capture tools hosted at Mayo Clinic.^14^, ^15^

The primary outcome was clinically significant CMV infection (csCMVi). The secondary outcome was all-cause mortality. CMV infection and disease were defined per published guidelines.^2^ csCMVi was defined as CMV disease or infection that necessitated antiviral therapy. Lymphopenia was defined as an ALC less than 0.61 × 10^9^/liter based on previous published literature.^6^, ^7^, ^16^

### Statistical analysis

Patient characteristics were summarized as median and interquartile range (IQR) for continuous variables and number and percentage for categorical variables. Kaplan-Meier curves were constructed to illustrate differences in cumulative incidence of csCMVi after heart transplantation between groups. Differences in csCMVi rates were compared using the log-rank test. Cox proportional hazards regression was used to analyze associations with csCMVi and all-cause mortality. Included variables in the csCMVi model were chosen a priori based on theorized associations or suspected confounding. The primary predictor of interest in the all-cause mortality model was csCMVi, and adjuster variables were suspected confounders with the primary predictor. All Cox models were stratified by the transplant center. Follow-up started on the date of transplantation and variables not known at baseline, such as development of csCMVi, were incorporated as time-dependent variables. ALCs were incorporated as a time-varying covariate with repeated measurements, where this variable was updated during follow-up with each successive measurement of ALC per patient from the date of transplantation until the date of either censoring or csCMVi. When analyzed as a continuous measure, a log2-transformation was utilized to account for right-skew and nonlinearity. As such, the interpretation of the hazard ratio (HR) for this ALC is the multiplicative change in hazard per 50% decrease in ALC. A sensitivity analysis was performed where ALC as a continuous measure was replaced by a binary lymphopenia variable (i.e., ALC < 0.61 × 10^9^/liter) in the multivariable Cox model. The proportionality assumption was assessed using Schoenfeld residuals. Due to nonproportionality of CMV serostatus in the CMV primary prophylaxis period compared to the later period, a 6-month interaction with time was incorporated for CMV serostatus in the multivariable model to generate separate HRs for this variable before and after 6 months of follow-up. Six-months was chosen to account for the period of primary valganciclovir prophylaxis. All analyses were performed using R version 4.2.2 (R Foundation for Statistical Computing, Vienna, Austria).

## Results

We included 553 adult patients who underwent heart transplantation during the study period (Table 1). Our cohort was predominantly male (N = 392; 70.9%), with a median age of 56.4 (IQR 45.8, 63.5) years. The most common indications for heart transplantation were nonischemic cardiomyopathy (N = 312; 56.4%) and ischemic cardiomyopathy (N = 168; 30.4%). Twelve (2.2%) patients underwent retransplantation. Antithymocyte globulin was used for induction immunosuppression in 468 (84.6%) of the cases; only a minority received induction immunosuppression with basiliximab (N = 40; 7.2%), alemtuzumab (N = 1, 0.2%), and methylprednisolone (N = 44, 8.0%).

**Table 1:** Cohort Characteristics of 553 Heart Transplant Recipients

Variables	D+/R– (N = 170)	R+ (N = 319)	D–/R– (N = 64)	Total (N = 553)
Age, years	56.8 (46.8, 63.8)	56.5 (47.0, 63.3)	52.1 (41.3, 61.6)	56.4 (45.8, 63.5)
Sex				
Female	50 (29.4%)	97 (30.4%)	14 (21.9%)	161 (29.1%)
Male	120 (70.6%)	222 (69.6%)	50 (78.1%)	392 (70.9%)
Heart transplant indication				
Amyloidosis	4 (2.4%)	11 (3.4%)	4 (6.2%)	19 (3.4%)
Ischemic cardiomyopathy	58 (34.1%)	92 (28.8%)	18 (28.1%)	168 (30.4%)
Nonischemic cardiomyopathy	92 (54.1%)	190 (59.6%)	30 (46.9%)	312 (56.4%)
Other	16 (9.4%)	26 (8.2%)	12 (18.8%)	54 (9.8%)
Retransplantation	1 (0.6%)	11 (3.4%)	0 (0.0%)	12 (2.2%)
Induction immunosuppression				
Alemtuzumab	0 (0.0%)	1 (0.3%)	0 (0.0%)	1 (0.2%)
Antithymocyte globulin	150 (88.2%)	262 (82.1%)	56 (87.5%)	468 (84.6%)
Basiliximab	11 (6.5%)	27 (8.5%)	2 (3.1%)	40 (7.2%)
No induction	9 (5.3%)	29 (9.1%)	6 (9.4%)	44 (8.0%)
Post-transplant renal replacement therapy	16 (9.4%)	23 (7.2%)	4 (6.2%)	43 (7.8%)
Absolute lymphocytes count at 1-month post-transplant, ×10^9^/liter	0.3 (0.2, 0.5)	0.4 (0.3, 0.6)	0.5 (0.3, 0.7)	0.4 (0.3, 0.6)
Lymphopenia at 1 month	140 (82.4%)	229 (71.8%)	42 (65.6%)	411 (74.3%)
Absolute lymphocytes count at 3-month post-transplant, ×10^9^/liter	0.5 (0.3, 0.7)	0.6 (0.4, 0.8)	0.7 (0.5, 0.9)	0.5 (0.3, 0.8)
Lymphopenia at 3 months	117 (68.8%)	178 (55.8%)	27 (42.2%)	322 (58.2%)
Acute rejection during follow-up	55 (32.4%)	102 (32.0%)	24 (37.5%)	181 (32.7%)

Data are N (%) or median (interquartile range).

Most heart transplant recipients were CMV seropositive before transplantation (N = 319; 57.7%). However, nearly one-third (N = 170; 30.7%) were CMV mismatched, with D+/R– status. There were 64 patients (11.6%) with CMV D–/R– status.

Lymphopenia was expectedly common after heart transplantation, with 74.3% (N = 411) of recipients being lymphopenic 1 month after transplantation and 58.2% (N = 322) being lymphopenic at 3 months post-transplantation. The median (ALC) at 1 and 3 months were 0.4 × 10^9^/liter and 0.5 × 10^9^/liter, respectively. Acute rejection occurred in 32.7% of patients during the follow-up period.

**Figure 1:**
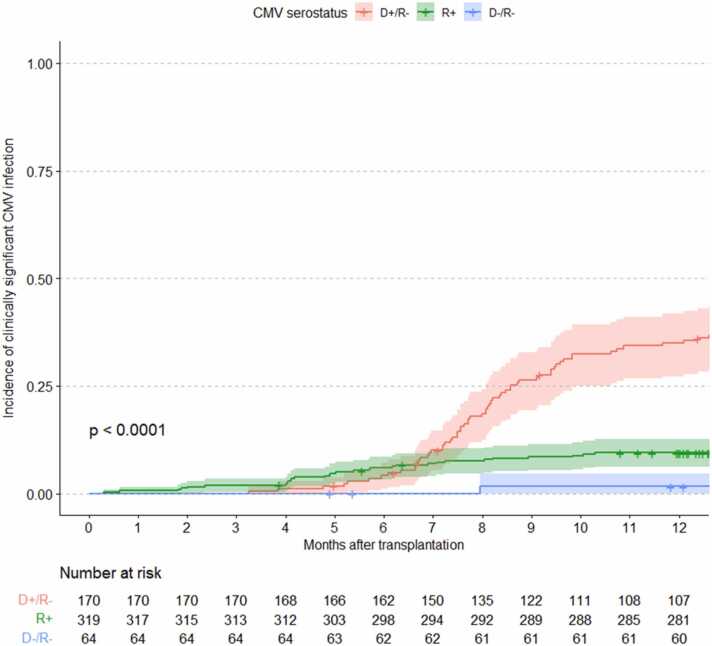
Kaplan-Meier curve of clinically significant CMV infection, grouped by serostatus.

csCMVi was observed in 101 patients (18.3%), including 24 patients with tissue-invasive CMV disease and 11 patients with CMV syndrome (Table 2). The most common site of tissue-invasive CMV disease was the gastrointestinal tract (57.1%). Pneumonitis occurred in 2 patients, and retinitis and disseminated disease was observed in 1 patient each. The median time from heart transplantation to csCMVi was 232 (IQR 181, 286) days (Figure 1). The median duration of treatment in mismatched patients was 51 (IQR 35-80) days and in R+ was 36 (IQR 27-55.5) days, for a total median duration of 44.5 (IQR 33-70) days.

**Table 2:** Characteristics of 101 Heart Transplant Recipients With Clinically Significant Cytomegalovirus Infection

Variables	D+/R– (N = 70)	R+ (N = 30)	D–/R– (N = 1)	Total (N = 101)
Time from transplant to diagnosis, days	248.5 (219.0, 297.8)	151.0 (123.0, 213.8)	242.0	232.0 (181.0, 286.0)
CMV disease	28 (40.0%)	7 (23.3%)	0 (0.0%)	66 (65.3%)
Gastrointestinal	15 (53.6%)	5 (71.4%)	0	20 (57.1%)
Nephritis and colitis	1 (3.6%)	0	0	1 (2.9%)
Pneumonitis	2 (7.1%)	0	0	2 (5.7%)
Retinitis	1 (3.6%)	0	0	1 (2.9%)
Viral syndrome	9 (32.1%)	2 (28.6%)	0	11 (31.4%)
Peak viral load, IU/ml	18,200.0 (3,280.0, 65,600.0)	4,815.0 (1,205.0, 11,950.0)	3,160.0	8,353.0 (2,655.0, 59,217.5)
Primary treatment agent				
Foscarnet	1 (1.4%)	1 (3.3%)	0 (0.0%)	2 (2.0%)
Ganciclovir	18 (25.7%)	7 (23.3%)	0 (0.0%)	25 (24.8%)
Ganciclovir + foscarnet	2 (2.9%)	0 (0.0%)	0 (0.0%)	2 (2.0%)
Valganciclovir	49 (70.0%)	22 (73.3%)	1 (100.0%)	72 (71.3%)
Treatment length, days	51.0 (35.0, 80.0)	36.0 (27.0, 55.5)	111.0	44.5 (33.0, 70.0)
Genotypic resistance	3 (4.3%)	1 (3.3%)	0 (0.0%)	4 (4.0%)
UL97	3 (4.3%)	1 (3.3%)	0 (0.0%)	4 (4.0%)
UL54	1 (1.4%)	1 (3.3%)	0 (0.0%)	2 (2.0%)

Data are N (%) or median (interquartile range).

Abbreviations: CMV, cytomegalovirus; D, donor; IU, international units; R, recipient.

In Table 3, we present the results of the multivariable Cox regression model for csCMVi. CMV D+/R– serostatus (compared to recipient seropositive status) was significantly associated with csCMVi after 6 months post-transplant (*p* < 0.001). Lower ALC and lymphopenia (i.e., ALC < 0.61 × 10^9^/liter) were each associated with a higher risk of csCMVi. When assessed as an interaction with serostatus, lower ALC (HR 1.48, 95% CI 1.23-1.79; *p* < 0.001) and lymphopenia (HR 3.21, 95% CI 1.52-6.75; *p* = 0.002) were both only significantly associated with csCMVi among CMV seropositive recipients, while these were not statistically significant among D+/R– patients. Acute rejection, induction immunosuppression with a lymphocyte depleting agent, age, and need for post-transplant renal replacement therapy were not associated with csCMVi.

**Table 3:** Multivariable Cox Regression Model for Clinically Significant CMV Infection

Variable	Hazard ratio (95% confidence interval)	*p*-value
Acute rejection^a^	0.78 (0.46, 1.33)	0.363
Absolute lymphocyte count (per 50% decrease), in R+^a^^,^^b^	1.48 (1.23, 1.79)	**<0.001**
Absolute lymphocyte count (per 50% decrease), in D+/R–a^,^^c^	1.18 (0.94, 1.47)	0.162
Lymphocyte depleting induction agent	0.85 (0.47, 1.53)	0.588
Age (per 10 years)	1.02 (0.86, 1.21)	0.802
Post-transplant renal replacement therapy	1.68 (0.85, 3.31)	0.133
CMV D+/R– before 6 months^d^	0.52 (0.21, 1.32)	0.168
CMV D–/R– after 6 months^d^	0.47 (0.06, 3.66)	0.471
CMV D+/R– after 6 months^d^	12.88 (6.76, 24.56)	**<0.001**

Abbreviations: CMV, cytomegalovirus; D, donor; R, recipient.

Bold values indicate *p* < 0.05. CMV serostatus was stratified using an interaction with time at 6 months to account for nonproportionality of the hazards between these 2 time periods, likely due to the use of post-transplant valganciclovir prophylaxis. D–/R– serostatus before 6 months was not reported due to this group not having any events.

a Analyzed as a time-dependent variable.

b A sensitivity analysis was performed where absolute lymphocyte count was dichotomized as a binary variable for lymphopenia, using ALC < 0.61 × 10^9^/liter as a cutoff. This showed HR 3.21 (95% CI 1.52-6.75; *p* = 0.002) in R+ patients.

c A sensitivity analysis was performed where absolute lymphocyte count was dichotomized as a binary variable for lymphopenia, using ALC < 0.61 × 10^9^/liter as a cutoff. This showed HR 1.42 (0.86-2.35; *p* = 0.176) in D+/R– patients.

d The reference for CMV serostatus is all R+ recipients. The serostatus before and after 6 months is based on serostatus at the time of transplantation.

During the median post-transplant follow-up of 2.3 (IQR 0.8-4.8) years, 60 people died. The median time to death after heart transplantation among those who died was 2.7 (IQR 1.0, 4.4) years. Four patients died and 1 was lost to follow-up within 6 months of transplantation. After adjusting for age, sex, acute rejection, retransplantation, and need for post-transplant renal replacement therapy, csCMVi was significantly associated with increased risk of mortality (HR 2.84, 95% CI 1.62-4.98; *p* < 0.001).

## Discussion

CMV in heart transplant recipients continues to be a prevalent opportunistic viral infection, associated with high morbidity, mortality, and cost of medical care.^13^, ^17^, ^18^, ^19^ In our cohort of heart transplant recipients, we analyzed the risk factors and outcomes of csCMVi, finding CMV mismatch serostatus, lower ALC, and lymphopenia to be associated with increased risk. Specifically, lower ALC and lymphopenia were only significant among seropositive recipients. csCMVi was also associated with increased post-transplant mortality, highlighting the poor outcomes from this infection.

The occurrence of csCMVi remains common in this cohort of heart transplant recipients and remains associated with high risk CMV donor and recipient serostatus. This risk was attenuated early after transplant, presumably due to primary valganciclovir prophylaxis.^20^ However, the most appropriate duration of prophylaxis continues to be debated, and the duration of prophylaxis varied among center-specific protocol without standardization.^21^, ^22^, ^23^ In our center, patients with high risk, mismatch serostatus (D+/R–) receive 6 months of valganciclovir prophylaxis, decreasing the risk of CMV reactivation during the prophylaxis period, and accounting for the low incidence of CMV episodes during this timeframe. Although CMV prophylaxis is helpful to decrease the risk of CMV reactivation, other host-specific factors play a key role, such as the degree of immunosuppression, duration of the prophylaxis, and the latency of the CMV in the host, conditions that are not always modifiable.^24^ After CMV prophylaxis is discontinued, the risk increases exponentially, emphasizing the need for better patient-specific and cost-effective strategies to mitigate this risk. The prolongation of CMV prophylaxis or the adaptation of post-prophylaxis CMV surveillance and pre-emptive therapy is also part of the protocols for management of CMV infection in solid organ transplant centers.^25^, ^26^, ^27^ Since the risk for CMV reactivation is dynamic, there have been efforts to build new testing strategies, such as CMV-cell mediated immunity assay,^28^ directed to estimate the CMV risk for each patient and individualize prophylaxis, and therapy approaches.^29^, ^30^, ^31^ Another strategy adapted is standardized patient education, aiming for early recognition of possible CMV infection or reactivation among CMV mismatched patients.^32^

Lymphopenia, usually defined <0.61 × 10^9^/liter, has been linked to increased risk for CMV reactivation in multiple studies and has been proposed as a readily accessible and cost-effective marker to guide risk assessment of CMV reactivation in Solid Organ Transplant recipients.^6^, ^7^, ^16^, ^30^, ^33^ A decrease in ALC was associated with a significantly increased risk of csCMVi in our recipient seropositive heart transplant population, supporting the possibility of using the inexpensive measure to estimate the risk for CMV reactivation. The association between decreased ALC seen in R+ patients may reflect the ability of the virus to reactivate when the patient has low immunity against CMV, especially CMV specific CD8+ T-cells. In mismatched patient such association was not seen, which may reflect the lack of CMV-specific immunity in these patients prevents the early recognition and reactivation of the virus, when compared with previously sensitized patients. Interestingly, more than lymphopenia as a binary marker, the downtrend of lymphocytes was associated with the development of csCMVi, and the uptrend associated with less development of csCMVi. Similar findings were reported recently where a decrease in ALC was associated with development of CMV infection.^16^ This suggests, besides simply assessing for an ALC cutoff, changes in ALC over time may be valuable in predicting risk for csCMVi. Given this association in the CMV R+ cohort, those with lymphopenia at time of planned prophylaxis discontinuation could then be considered for either postprophylaxis CMV monitoring or even prolongation of prophylaxis.

csCMVi has also been linked to increased mortality in heart and other Solid Organ Transplant recipients,^18^, ^19^, ^20^, ^34^, ^35^ especially patients who develop severe CMV disease. The exact contribution of CMV to the overall cause of mortality in heart transplant recipients is difficult to establish, especially since more than being directly associated with the infection, may reflect the conglomerate of other events surrounding and episode of CMV in a heart transplant recipient. The development of other opportunistic infections along with the CMV episode, overimmunosuppression, or decrease on immunosuppressants leading to rejection are other possible contributing factors to the overall increased in mortality, and this study adds to the existing evidence on this association. Increase in mortality further suggests the need for novel preventive approaches to decrease the episodes of CMV reactivation/infection in heart transplant recipients to decrease this risk.

Association between CMV and allograft rejection has been suggested by several studies and has been linked with increased mortality and need for retransplantation.^36^, ^37^, ^38^ In our cohort, heart transplant recipients who received treatment for allograft rejection were at risk for CMV infection routinely received antiviral prophylaxis, which may explain why no association was seen between allograft rejection and subsequent CMV infection. Induction with lymphocyte depleting agents and use of high doses of steroids have classically been associated with an increased risk for development of csCMVi, in our cohort, such association was not found.^4^, ^39^, ^40^, ^41^, ^42^ The lack of association between these factors and the development of csCMVi is not totally clear, but the improvement in prophylaxis use, as well as monitoring in high-risk patients, could account for the lack of association found in this cohort. Any association between either lymphocyte-depleting agents and high-dose corticosteroids and subsequent csCMVi may also be mediated through decreases in ALC and lymphopenia.

Our study has several limitations worth noting. The main limitation of our study is its retrospective nature, and it is subject to intrinsic sources of confounding and bias. Post-transplant CMV monitoring also varied by serostatus, and closer monitoring of CMV mismatched patients may have contributed to their higher incidence. ALC overtime was analyzed, but ALC immediately before csCMVi episode was not included as separate analysis. We used ALC measurements drawn as part of routine clinical practice, and these may have been drawn at different time points/different frequency between patients. A thorough assessment of post-transplant renal replacement therapy and csCMVi episodes was limited due to the small number of patients who required renal replacement therapy. Large-scale studies studying the association between this variable and the impact on development of csCMVi are needed. Most patients received a lymphocyte-depleting agent for induction immunosuppression, limiting our ability to assess patients without depleting induction immunosuppression. Finally, there was only 1 CMV D–/R– patient who developed csCMVi, significantly limiting the ability to assess risk factors in this group.

### Future perspective

csCMVi is a prevalent opportunistic infection affecting transplant recipients, including heart transplantation recipients. As this study shows, the most important risk factor is CMV serostatus mismatch and decrease in ALC among CMV seropositive recipients. Patients with csCMVi remain at increased risk of mortality. Large-scale studies associating csCMVi and long-term outcomes in heart transplant recipients, including long-term allograft function, are needed. Given the significant csCMVi noted in current heart transplantation era despite of antiviral prophylaxis, supplemental strategies need to be considered to lessen CMV infection and related complications, particularly among CMV mismatched recipients and seropositive recipients with lymphopenia. The effectiveness of pre-emptive therapy approach in heart transplant recipients needs further exploration, and more studies assessing the effectiveness and logistics for this approach would be helpful determining the impact in outcomes, including studies showing outcomes with newer approaches, such as letermovir and maribavir.

## Disclosure Statement

R.R.R. receives research support (funds provided to the institution) from Gilead, Regeneron, Roche, the MITRE corporation, and Nference, Inc. R.R.R. serves on the advisory board for Glaxo Smith Kline and on the Data Safety Monitoring Board for Novartis. None of these entities have provided support for this current study. All authors have no conflicts of interest to report.

This project was supported by Grant Number UL1 TR002377 from the 10.13039/100006108National Center for Advancing Translational Sciences (NCATS). Its contents are solely the responsibility of the authors and do not necessarily represent the official views of the NIH.
